# Chemometric analysis combined with FTIR spectroscopy of milk and Halloumi cheese samples according to species’ origin

**DOI:** 10.1002/fsn3.1603

**Published:** 2020-05-12

**Authors:** Maria Tarapoulouzi, Rebecca Kokkinofta, Charis R. Theocharis

**Affiliations:** ^1^ Department of Chemistry University of Cyprus Nicosia Cyprus; ^2^ State General Laboratory Nicosia Cyprus

**Keywords:** authenticity, chemometrics, FTIR spectroscopy, Halloumi cheese, milk, species’ origin

## Abstract

Food adulteration is an issue of major concern, as numerous foodstuffs and beverages do not follow their labeling. Our research interest is in the field of authenticity of dairy products and particularly cheese. Adulteration of dairy products is a well‐known phenomenon, and there are numerous published studies specifically on the authenticity of cheese. In fact, substitution of a portion of fat and/or proteins, adulteration with milk of other species’ origin, and mislabeling of ingredients are some of the main issues that the science of dairy products’ authenticity is regularly facing. Discrimination of dairy products can be determined through several chemical or microbiological methods as presented in the literature. In addition, chemometric analysis is an important tool for interpretation of a huge load of measurements. The aim of this study is to discriminate between various milk samples, which is the primary ingredient of dairy products. Milk samples with different trademarks were analyzed. That data was combined with Halloumi cheese samples for chemometric discrimination of species’ origin. The innovative point of this study is the fact that it is the first time that a research study related to dairy products includes Halloumi cheese which is a traditional Cypriot cheese, not well‐studied until now. The first step of the methodology was the freeze‐drying via lyophilization of the samples. Fourier transformed infrared spectroscopy (FTIR) was chosen for their chemical characterization. Moreover, interpretation of the measurements was carried out by chemometric analysis using SIMCA software. For this study, FTIR data combined with chemometrics have given a very good discrimination of the samples according to their species’ origin. Chemometric methods such as PCA and OPLS‐DA have been used with great success. In the future, this model will be studied regarding geographical origin of the samples.

## Graphical abstract

Characterization of the traditional Cypriot cheese Halloumi. Examination of ways to detect the animal origin of the milk used in the final product. Chemometric analysis of spectroscopic data

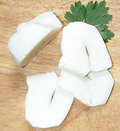

## 1 INTRODUCTION

Milk is used as an ingredient in many food products for human nutrition. The characteristics of many dairy products are related to the quality and to the species’ origin of the milk used, as mixtures of different kinds of milk, at specific ratios, contribute in giving special properties to the final product. Milk quality is also important in the production of all types of cheese. The commercial value of these dairy products is often determined by the exact composition of the milk mixture used (Lamanna, Braca, Di Paolo, & Imparato, 2011). Goat and sheep milk are of higher value than cow milk and are used to produce a variety of specialty cheeses.

The fact that milk and dairy products are consumed by large segments of society is the reason that motivates unscrupulous producers to proceed to adulterations in order to maximize their profit with negative effects on product quality (Cirak, Icyer, & Durak, 2018). Adulterations of either goat or sheep milk with cow milk result in nonauthentic milk products (Maudet & Taberlet, 2001; Nicolaou, Xu, & Goodacre, 2010). The European Union has legislation in place for the correct display of the constituents of dairy products protecting their authenticity (European Commission, 2001).

Milk is the only dairy food whose determination of adulteration of milk species is the potential drawback of biological techniques. Some authors state that during the analysis of milk with biological techniques, proteolysis, and heat denaturation can cause the loss of antibody‐specific epitopes (Hurley, Elyse Ireland, Coleman, & Williams, 2004; Nicolaou et al., 2010). In addition, biological techniques, such as DNA based ones, can be impractical for routine industrial use, and quantification aspects may be hindered by environmental factors such as mastitis, or by milk processing factors such as milk heat treatment (Bania, Ugorski, Polanowski, & Adamczyk, 2001; López‐Calleja et al., 2005; Cheng, Chen, & Weng, 2006; Mašková & Paulíčková, 2006; Nicolaou et al., 2010). Heat treatment is used as an integral part of halloumi production. In addition, sodium dodecyl sulfate polyacrylamide gel electrophoresis (SDS‐PAGE) is considered as a time‐consuming approach, which must be followed by in‐gel digestion to identify the marker peptides of milk and to discriminate the samples regarding species’ origin (Calvano, De Ceglie, Monopoli, & Zambonin, 2012).

To differentiate milk samples according to species’ origin, various chemical analytical techniques have been used from all the well‐known categories in food authentication (Medina, Perestrelo, Silva, Pereira, & Câmara, 2019), such as chromatographic, spectroscopic, mass spectrometry techniques, and electronic sensors. Moreover, chemometrics and spectroscopic techniques are widely used in food science nowadays (De Luca et al., 2019; Huang et al., 2020; Li, Li, Li, Liu, & Wang, 2019; Mabood et al., 2019; Sánchez‐Rodríguez, Sánchez‐López, Marinas, Urbano, & Caridad, 2019; Velázquez Ríos et al., 2019). A huge number of publications exists for discrimination of milk or cheese samples and they combine the spectroscopic technique FTIR (Fourier transformed infrared spectroscopy) and chemometrics regarding species’ origin (Cirak et al., 2018; Nicolaou et al., 2010; Pappas et al., 2008; Terouzi, Omari, Boutoial, & Oussama, 2016). In addition, various wavenumber ranges have been chosen among the studies performed with FTIR, making the discrimination of the samples in this study more challenging.

Halloumi cheese is a semi‐hard to hard, elastic, rindless, and easily sliced cheese. It does not belong in the category of rind or cheese with holes (eyes). Gibbs, Morphitou, and Savva (2004) stated that Halloumi cheese contains more calcium and less cholesterol than Cheddar cheese. In addition, it is the only cheese in the world which can be consumed uncooked, fried, grilled, baked, or boiled (Gibbs et al., 2004). It has a white to yellow color, which may fade depending on the milk species’ origin. Goat and sheep milk gives a white color; however, cow milk turns Halloumi into a yellowish cheese (Kaminarides, Rogoti, & Mallatou, 2000; Lteif, Olabi, Baghdadi, & Toufeili, 2009; Robinson, Haddadin, & Abdullah, 1991). Halloumi cheese is the primary traditional cheese in Cyprus, but so far, is neither a PDO‐, nor a PGI‐cheese. The traditional recipe of Halloumi cheese demands the use of goat and/or sheep milks; however, fully cow milk is often used by the large dairy industry. In addition, local Halloumi cheesemakers use unique empirical conditions of processing and milk proportions, thus product characteristics may vary from dairy to dairy throughout the island. Further variation may be expected when it is made outside Cyprus where different standards may apply. The consumers of Halloumi cheese in Cyprus prefer the homemade products rather than industrial ones; however, prices of homemade Halloumi cheese vary widely.

The species’ origin of milk must be labeled on the package of the product (Moatsou, Hatzinaki, Psathas, & Anifantakis, 2004). The substitution of goat or sheep with cow milk in cheese can be determined by using the European reference method (European Commission Regulation, 1996). The method is based on isoelectric focusing of γ‐caseins after treatment of cheese casein fraction with plasmin. Nevertheless, the substitution of sheep with goat milk cannot be determined with this method, and this is also a common issue in the field of food adulteration (Moatsou et al., 2004; Recio et al., 2004). In the 1980s, a national legislation was voted in Cyprus for protecting the dairy industry, which it stated that only a substantial proportions of goat and/or sheep milk must be contained within any cheese sold as Halloumi, without any fixed percentages or thresholds (Welz, 2017). This was changed in 2012 when a minimum of fifty‐one percent milk from sheep and goat became mandatory, as stated in the Official Gazette of the Republic of Cyprus, 30 November 2012, Issue No 4628, p. 4786 (Welz, 2017).

The aim of this study is to discriminate between various milk samples. Milk samples with different trademarks were analyzed. The main goal of this study is the development of a method, based on spectroscopy, capable of determining, and identifying the adulteration in Halloumi cheese due to mislabeling regarding species’ origin of milk. It is the first time that a research study related to dairy products includes Halloumi cheese, which is not well‐studied until now. The first step of the methodology was the freeze‐drying process with lyophilization of the samples. FTIR was chosen in order to chemically characterize the samples. The advantages of FTIR spectroscopy are that assessments can be accurately reproduced and analyzed without any experimentation and without damaging the sample. It can give results even with a minimum sample amount and does not require any additional substances or chemicals; thus, it reduces the cost and time of analysis (Cirak et al., 2018; Ketty et al., 2017; Nicolaou et al., 2010; Pappas et al., 2008; Terouzi et al., 2016). Moreover, interpretation of the measurements was done with chemometric analysis based on SIMCA software. Chemometric methods such as PCA and OPLS‐DA have been used with great success.

## 2 MATERIALS AND METHODS

### 2.1 Samples analyzed

Twenty‐eight milk samples and seventy‐four Halloumi cheese samples of different species’ origin were purchased from local supermarkets in Cyprus and were analyzed to compare their infrared spectra regarding species’ origin.

### 2.2 Lyophilization

A Christ, Alpha 1–2 freeze drier was used. The condenser temperature was 233 K and the final pressure in the drying chamber 3 mPa. The freeze‐drying procedure for 3 ml or 5 g of each milk or Halloumi cheese sample required 8 or 5 hr, respectively, to be completed and the residue after homogenization was used for FTIR measurements.

### 2.3 FTIR Studies

The FTIR spectra were measured in duplicate on a Shimadzu Fourier Transform ‐ 8,900 Spectrometer instrument employing a KBr beam splitter. Samples were recorded as pressed KBr pellets. Twenty scans were co‐added at a normal resolution of 8 cm^−1^ in the 4,000–400 cm^−1^ region. The samples were recorded against a background of air to minimize the interference due to carbon dioxide and water vapor in the atmosphere. The region between 2,700 and 1900 cm^−1^ was removed prior to multivariate data analysis because the absorbance of carbon dioxide (CO_2_) is included in this region, and specifically the absorption at 2,360 cm^−1^ known to be caused by atmospheric carbon dioxide (Ketty et al., 2017). In addition, this region may contribute with more noise than chemical information, due to lack of molecular vibrations in the particular parts of the spectra.

### 2.4 Data analysis

All the spectra were imported into Excel before chemometrics. Multivariate statistical analysis including unsupervised principal component analysis (PCA), supervised orthogonal partial least‐squares discriminant analysis (OPLS‐DA) was performed using SIMCA software (version 15.0.2; Umetrics; Sweden). Prior to fitting OPLS‐DA, a preliminary PCA was performed for data overview. Scaling to unit variance (UV) and mean‐centering were applied before analysis. The ellipse in the plots defines Hotelling's T2 confidence region, which is a multivariate generalization of Student's *t* test and provides a 95% confidence interval for the observations. The number of the important components which have been chosen is given with the symbol A. R*^2^*X and R*^2^*Y* *represent the cumulative modeled variation, explaining the quality of the model, and Q*^2^* is an estimate of model predictive ability, calculated by cross‐validation analysis of variance (CV‐ANOVA). R*^2^*X*, *R*^2^*Y, and Q*^2^ *values (not less than 0.5) suggested a robust model with predictive reliability (Yang et al., 2016). The difference between R^2^X (cum) and Q^2^ (cum) must be lower than 0.2–0.3 (Eriksson et al., 2006; Fotakis et al., 2016). Regarding the predictability of the OPLS‐DA models, the misclassification percentage was calculated. Furthermore, on the cross‐validation (CV) score plot, if some samples cross over to the other side, this indicates that their class assignment is uncertain. In addition, model validation was performed by permutation tests repeated 100 times. To indicate the validity of the original models, both the blue and green regression lines of the Q^2^ and R^2^ points, respectively, should intersect the vertical axis at, or below, zero. Based on the CV‐ANOVA results, a hypothesis test takes place where “Null Hypothesis” indicates that population means of the different appraisers are equal, and “Alternate Hypothesis” shows that one of the means is not the same. Larger values of *F*_statistic_ than the *F*_critical_ indicate that the difference of the mean of the samples is large compared to the dispersion of the observations within each database, and therefore, the null hypothesis should be rejected, and the alternate hypothesis is considered important at the 5% level. Similarly using *p*‐value approach, which should be less than 0.05.

Lastly, other dataset was used in conjunction with the model constructed here to gauge the repeatability of the model in this study.

## 3 RESULTS AND DISCUSSION

### 3.1 Characterization of FTIR spectra of milk samples

**FIGURE 1:**
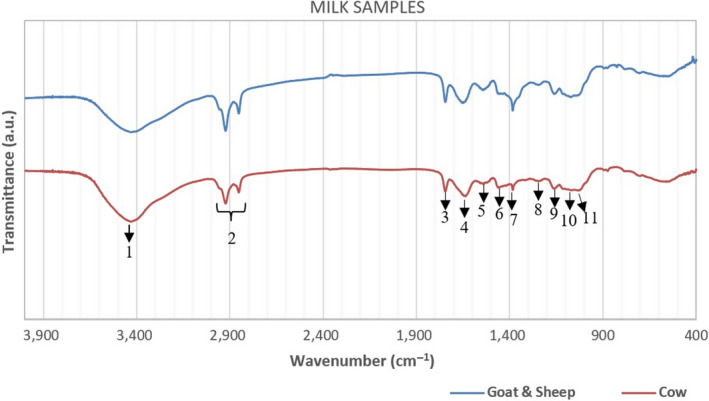
Representative samples of milk database regarding species’ origin

The differences between goat–sheep against cow milk samples are depicted in Figure 1. Between the two origin samples of Figure 1 clear differences can be specifically seen at the bands as follows:

- 1:3,450 cm^−1^: it is related to –ΟΗ stretching in hydroxyl groups,
- 2:2,930 – 2,850 cm^−1^: it is associated with C‐H bending in fatty acids,
- 3:1745 cm^−1^: it is correlated to the degree of sugars carboxyl methyl esterification,
- 4:1683 cm^−1^ (broad peak): it has been assigned to the carbonyl (C = O) stretching (amide I), and it may be overlapped with the broad and weak peak at 1644 cm^−1^ which is correlated with the nonremoved water (bending vibration),
- 5:1548 cm^−1^ and 6:1,453 cm^−1^: they correspond to the N–H bending with the contribution from C–N stretching (amide II),
- 7:1,397 cm^−1^: it is associated with C‐H bending of esters and aliphatic chains of fatty acids,
- 8:1,245 cm^−1^: it corresponds to C‐H bending and C–N stretching with the contribution from N–H bending (amide III),
- 9:1,168 cm^−1^: it is due to –NH_2_ deformation,
- 10:1,100 cm^−1^: it is assigned to COH bending and C–C stretching with the contribution from OH bending (Pappas et al., 2008),
- 11:1,100 – 1,060 cm^−1^: it is associated with O = P–O (phosphate groups stretch) covalently bound to casein proteins can be also observed in milk samples (Etzion, Linker, Cogan, & Shmulevich, 2004).

Variations between samples depend on the respective functional groups, and therefore, it was possible to construct chemometric models.

### 3.2 Characterization of FTIR spectra of Halloumi cheese

**FIGURE 2:**
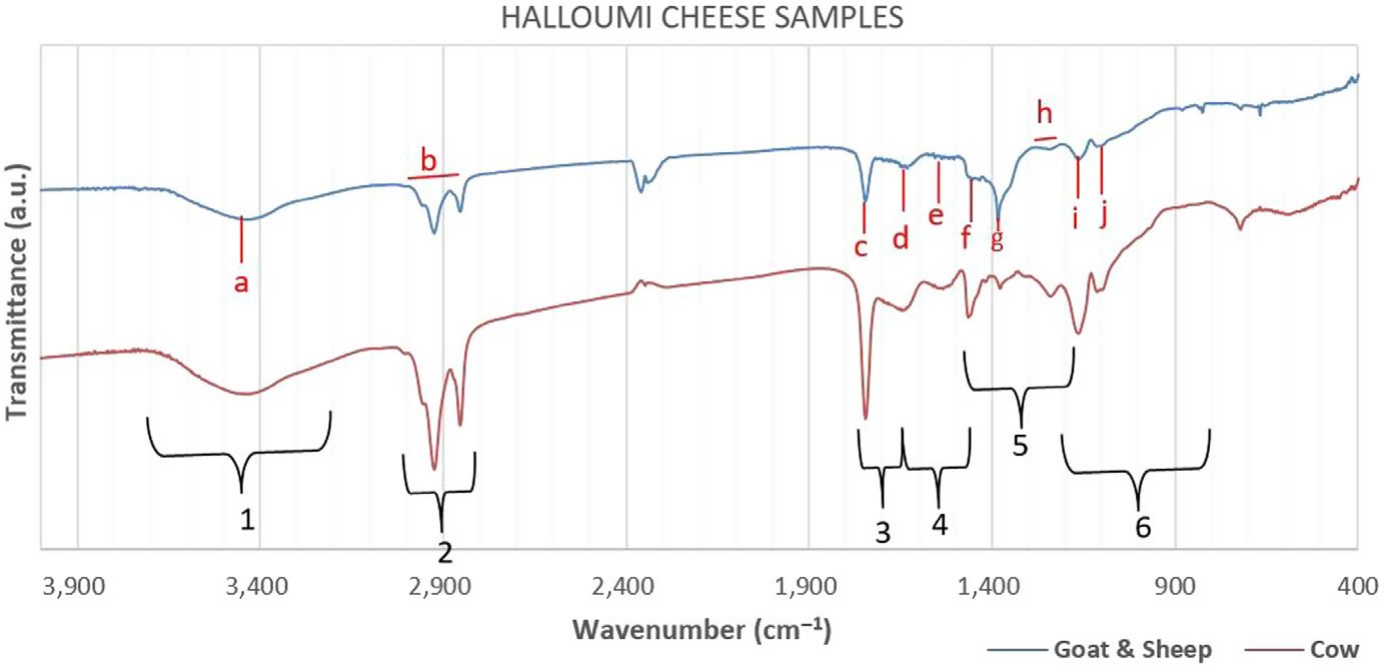
Representative samples of Halloumi cheese database regarding species’ origin, where black shapes and numbers (1–6) show the general regions with differences between the species’ origins, and red letters (a–j) show the specific bands which are different between the two origins

In general, a spectrum obtained from a cheese can have the following characteristic bands, as shown in Figure 2:

- 1:3,700 – 3,200 cm^‐1^: –ΟΗ and ‐NH stretching in proteins,
- 2:3,000 – 2,800 cm^‐1^: C‐H stretching in fatty acids,
- 3:1,750 – 1,650 cm^‐1^: ‐C = O of fatty acids and esters,
- 4:1,650 – 1,450 cm^‐1^: ‐C = O and ‐NH from amides Ι and ΙΙ of proteins resulting from different combinations of vibrations in the peptide bonds and the secondary structure of casein protein. The amide I vibration is caused primarily by the stretching of the C = O bonds, and the amide II vibration is caused by deformation of the N–H bonds and stretching of the C–N bonds. The amide I vibration is measured in the range from 1,650 to 1,550 cm^−1^ and the amide II region from 1,550 to 1,450 cm^−1^,
- 5:1,460 – 1,150 cm^‐1^: esters and aliphatic chains of fatty acids,
- 6:1,200 – 800 cm^‐1^: ‐C = O from polysaccharides and C = C stretching of acids (Nicolaou & Goodacre, 2008; Subramanian, Alvarez, Harper, & Rodriguez‐Saona, 2011).

When the results of the measurements are observed, it is seen that the spectrums of cow milk and goat–sheep milk yield absorption values within more or less the same areas. These particular areas, too, can be used for classifying the species’ origin of milk based on different relative intensity of absorption found for respective milk types, as shown in Table 1.

**TABLE 1:** Type of relative intensity of absorption for important subregions regarding species’ origin

No.	Subregion	Relative intensity of absorption	
		Cows product	Goats and sheep product
1	3,700–3,200 cm^−1^	strong	medium
2	3,000–2,800 cm^−1^	strong	medium
3	1,750–1,650 cm^−1^	medium	strong
4	1,650–1,450 cm^−1^	medium	strong
5	1,460–1,150 cm^−1^	medium	strong
6	1,200–800 cm^−1^	strong	medium

The different regions between goat–sheep and cow products summarized in Table 1 are also depicted in Figure 2 for Halloumi cheese samples. Between the two origin samples of Figure 2 clear differences can be specifically seen at the bands (a–h) as follows:

- a: 3,450 cm^−1^: it is related to –ΟΗ stretching in hydroxyl groups,
- b: 2,930 – 2,850 cm^−1^: it is associated with C‐H bend in fatty acids,
- c: 1745 cm^−1^: it is correlated with the degree of sugars carboxyl methyl esterification,
- d: 1683 cm^−1^ (broad peak): it has been assigned to the carbonyl (C = O) stretching (amide I), and it may be overlapped with the broad and weak peak at 1644 cm^−1^ which is correlated with the nonremoved water (bending vibration),
- e: 1548 cm^−1^ and f: 1,453 cm^−1^: it corresponds to the N–H bending with the contribution from C–N stretching (amide II). More specifically, small differences between the two spectra can be observed from amino acid side chain vibrations due to tyrosine at about 1515 cm^‐1^, phenylalanine at about 1,498 cm^‐1^, proline at about 1,453 cm^‐1^, and sometimes a small absorption at 1438cm^‐1^.
- g: 1,397 cm^−1^: they are associated with C‐H bending of esters and aliphatic chains of fatty acids,
- h: 1,245 – 1,243 cm^−1^: it corresponds to C‐H bending and C–N stretching with the contribution from N–H bending (amide III),
- i: 1,168 cm^−1^: it is due to –NH_2_ deformation, and
- j: 1,100 cm^−1^: they are assigned to COH bending and C–C stretching with the contribution from OH bending (Elbassbasi, Kzaiber, Ragno, & Oussama, 2010; Pappas et al., 2008).

### 3.3 Setting‐up a database for milk samples

The total number of milk samples was 26, since samples M3 and M12 have been removed from the dataset after the first overview plot produced by PCA (not shown here). Sample M3 was the only outlier sample and M12 was the only vegetarian sample, and both of them were disturbing the process of chemometric analysis. M12 fell in the class of goat–sheep origin as it is a vegetarian sample made of plant fats, and bearing in mind that goat and sheep are fed outside the farms, grazing plants, this may be the reason explaining its clustering with the samples of goat–sheep origin. Regarding M3, it can be said that outlier samples’ occurrence is usually mentioned for reasons like a strong change in the sample composition, incorrect sample preparation or storage and instrumental artifacts. A loading plot (not shown here) was produced, and it showed that only the subregions of 3,500–3,300 and 2,900–2,800 cm^−1^ should proceed in chemometric analysis, which correspond to ‐OH stretching in hydroxyl groups and C‐H bending in fatty acids, respectively. The fingerprint area at approximately 1,600–650 cm^−1^ was expected to be significant according to the data in Table 1; however, the loading plot showed that it was not.

**FIGURE 3:**
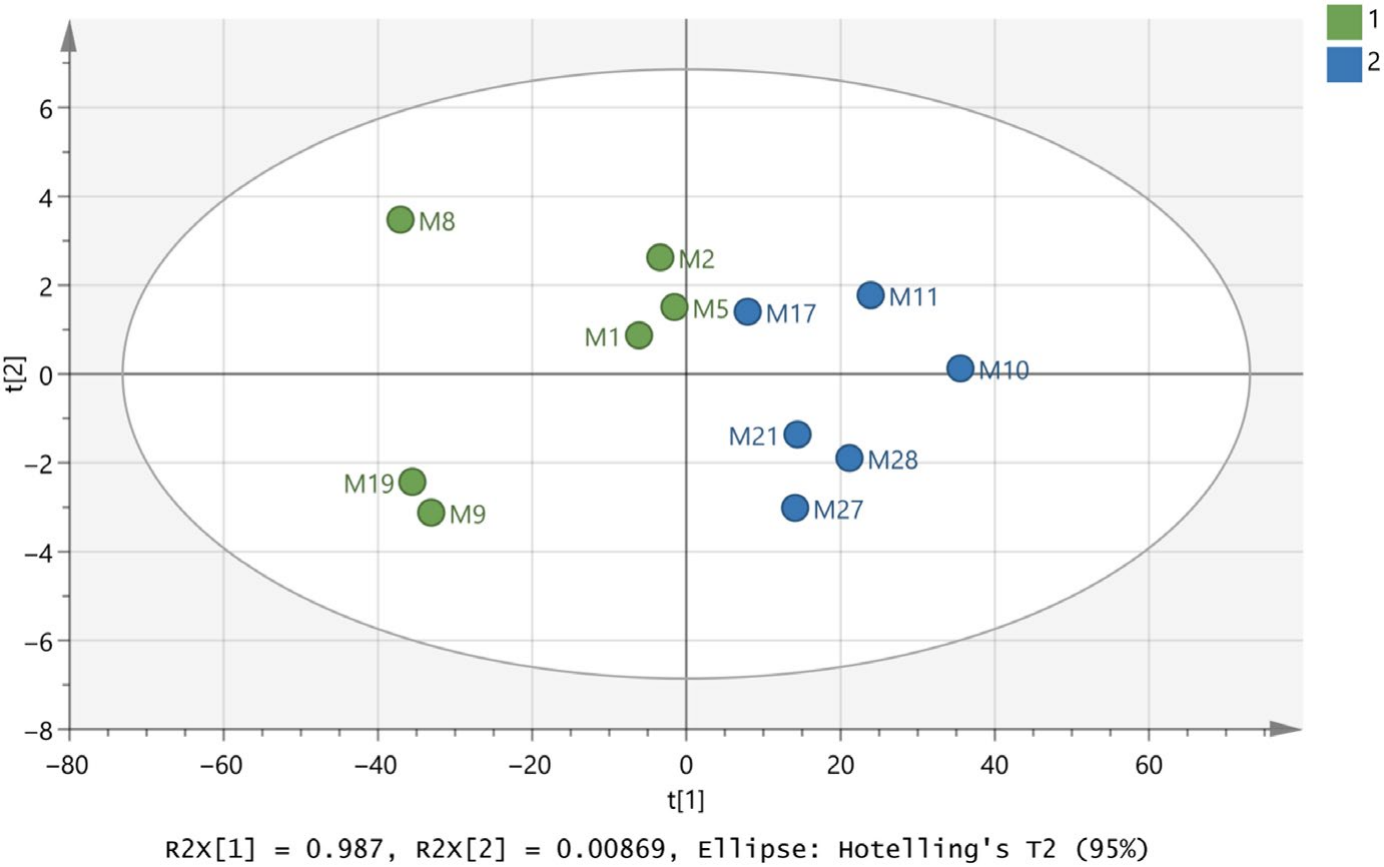
Score scatter plot (t2/t1) from PCA modeling of the training set regarding species’ origin of milk samples by using the subregions 3,500–3,300 and 2,900–2,800 cm^‐1^ from FTIR spectra. A = 2 components, R^2^X(cum) = 0.995, Q^2^(cum) = 0.993

**FIGURE 4:**
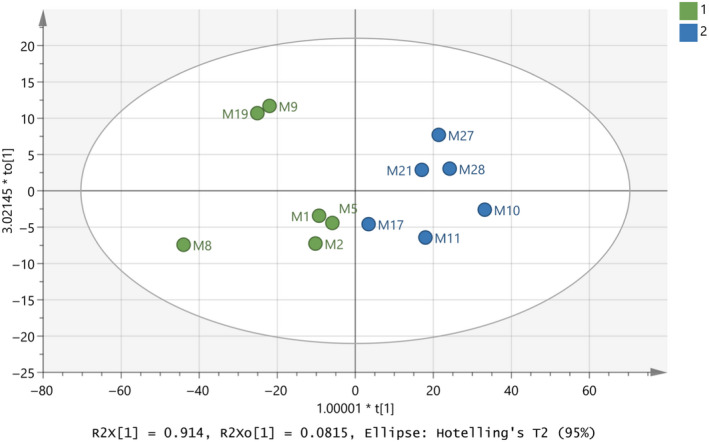
Score scatter plot (to1/t1) from OPLS‐DA modeling of the training set regarding species’ origin of milk samples by using the subregions 3,500–3,300 and 2,900–2,800 cm^−1^ from FTIR spectra. A = 1+1 components, R^2^X(cum) = 0.995, R^2^Y(cum) = 0.737, Q^2^(cum) = 0.619

After that, a training set was set up with the twelve representative (authentic) milk samples for each of the two classes, namely cow origin for class 1 and goat–sheep origin for class 2. Figures 3 and 4 present the constructed model for this attempt modeled with PCA and OPLS‐DA, respectively. In both cases, cow samples are located on the left‐hand side, while goat–sheep samples on the right‐hand side of the graph. For PCA, high values for R^2^X(cum) = 0.995 and Q^2^(cum) = 0.993 have been calculated, as well as OPLS‐DA has given R^2^X(cum) = 0.995, R^2^Y(cum) = 0.737, and Q^2^(cum) = 0.619. The difference between R^2^X (cum) and Q^2^ (cum) is relatively low and close to 0.3.

In addition, misclassification table for the OPLS‐DA modeling is depicted in Table 2, and it also shows 100% percentage of correct classification, while the low Fischer value of *p* < .05 emphasizes the statistical importance of the training set of this model.

**TABLE 2:** Misclassification table from OPLS‐DA modeling of the training set regarding species’ origin of milk samples, where 1: Cows samples and 2: Goats and sheep samples

	Members	Correct	1	2
1	6	100%	6	0
2	6	100%	0	6
Total	12	100%	6	6
Fisher's prob.	0.0011			

**FIGURE 5:**
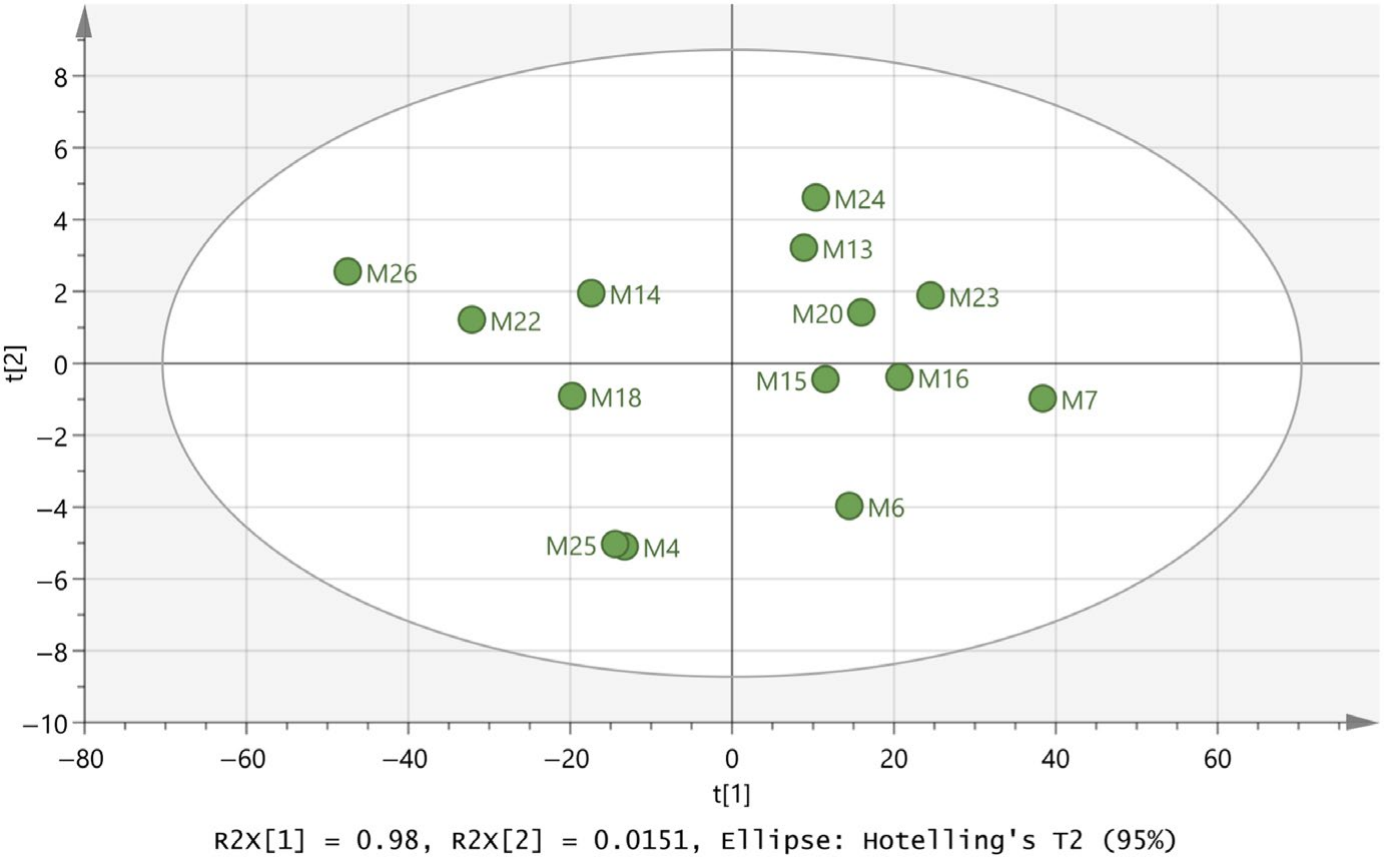
Score scatter plot (t2/t1) from PCA modeling of the test set regarding species’ origin of milk samples by using the subregions 3,500–3,300 and 2,900–2,800 cm^−1^ from FTIR spectra. A = 2 components, R^2^X(cum) = 0.996, Q^2^(cum) = 0.994

Moreover, a test set was prepared with the fourteen samples excluded from the training set as they had unknown animal origin. PCA was the only available chemometric method of analysis because the classes of the samples were unknown in the test set. Figure 5 depicted the model of the test set, and it can be seen that six samples (i.e., M4, M14, M18, M22, M25, and M26) are clearly grouped on the left‐hand side, and eight samples (i.e., M6, M7, M13, M15, M16, M20, M23, and M24) on the right‐hand side of the graph. This observation may lead to the conclusion that, similar with the training set, cow samples may be located on the left‐hand side, while goat–sheep samples on the right‐hand side of the graph. The values of R^2^X(cum) = 0.996 and Q^2^(cum) = 0.994 show the importance of the model produced for the test set, as they are both close to 1.

**FIGURE 6:**
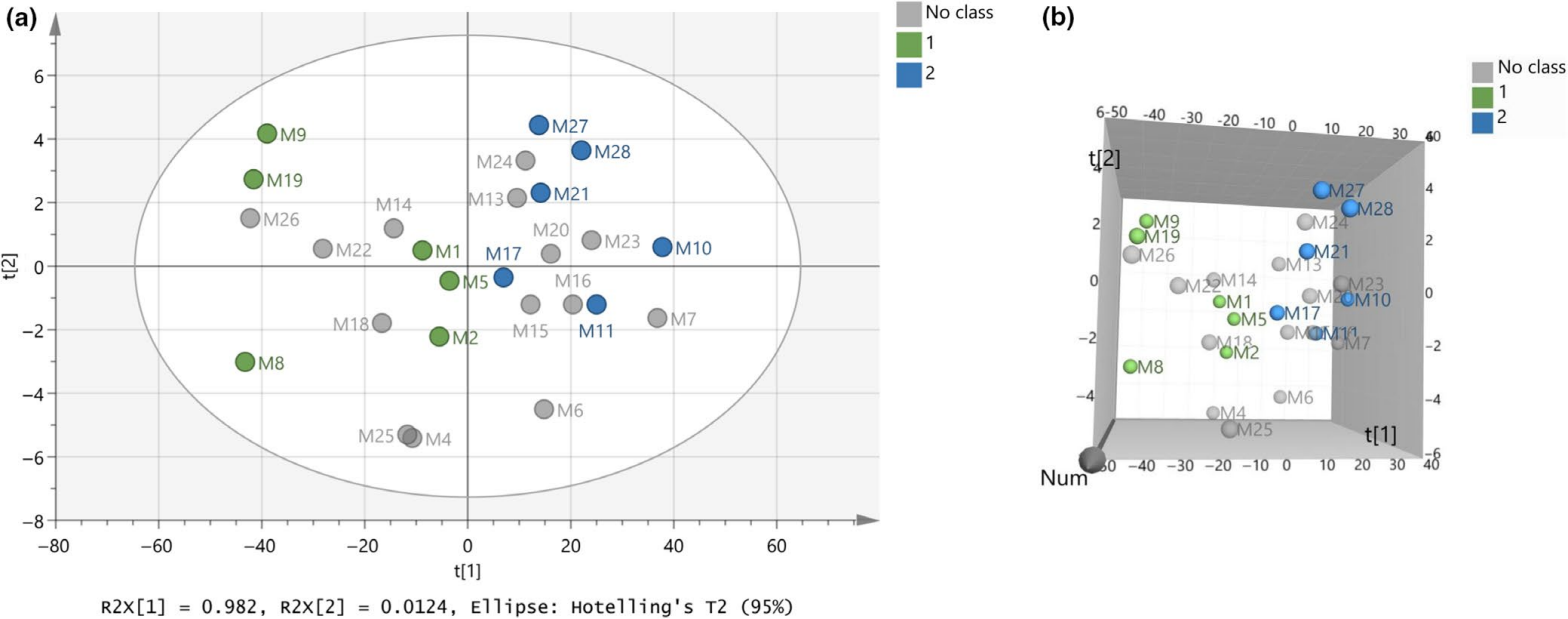
(a) Score scatter plot (t2/t1) from PCA modeling of the training merged with the test set regarding species’ origin of milk samples by using the subregions 3,500 – 3,300 and 2,900 – 2,800 cm^‐1^ from FTIR spectra. A = 2 components, R^2^X(cum) = 0.995, Q^2^(cum) = 0.994, (b) 3D presentation of the score scatter plot (t2/t1/Num) of (a)

After that, merging of the two sets (i.e., training and test sets) had to take place. Figure 6a,b show the model constructed after merging the two sets, whereas in the training set, green is the color of cow samples, blue is for goat–sheep samples, and gray is the color for all the samples presented in the test set. By comparing the model of test set (Figure 5) and the merged set (Figure 6a), it can be observed that no sample of the test set change side in the ellipse, and the six samples (i.e., M4, M14, M18, M22, M25, and M26) are still grouped on the left‐hand side, and the eight samples (i.e., M6, M7, M13, M15, M16, M20, M23, and M24) are still on the right‐hand side of the graph. It is now clear that left‐hand side location has the cow samples and right‐hand side the goat–sheep samples.

**FIGURE 7:**
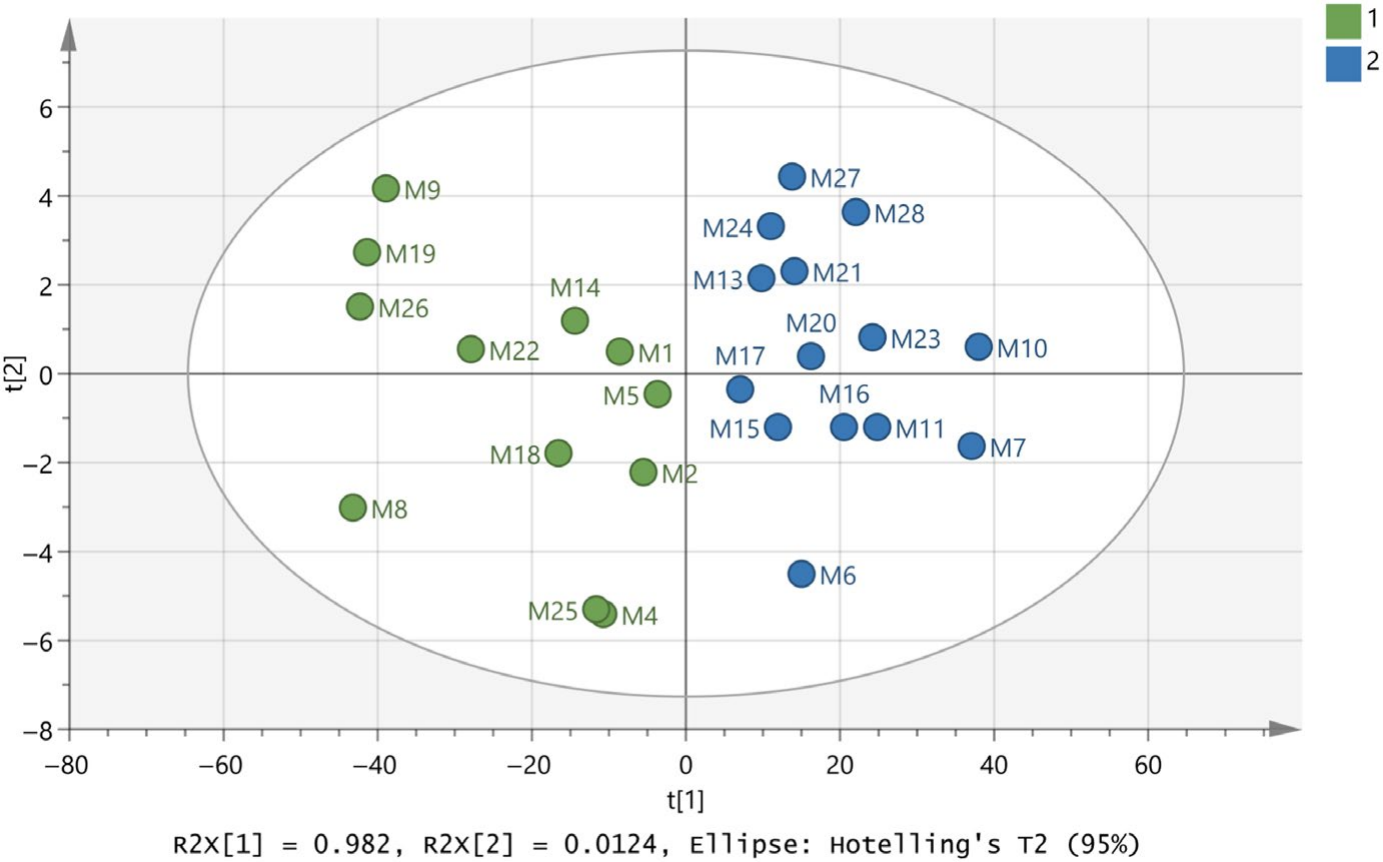
Score scatter plot (t2/t1) from PCA modeling of the final database regarding species’ origin of milk samples by using the subregions 3,500 – 3,300 and 2,900 – 2,800 cm^‐1^ from FTIR spectra. A = 2 components, R^2^X(cum) = 0.995, Q^2^(cum) = 0.994

**FIGURE 8:**
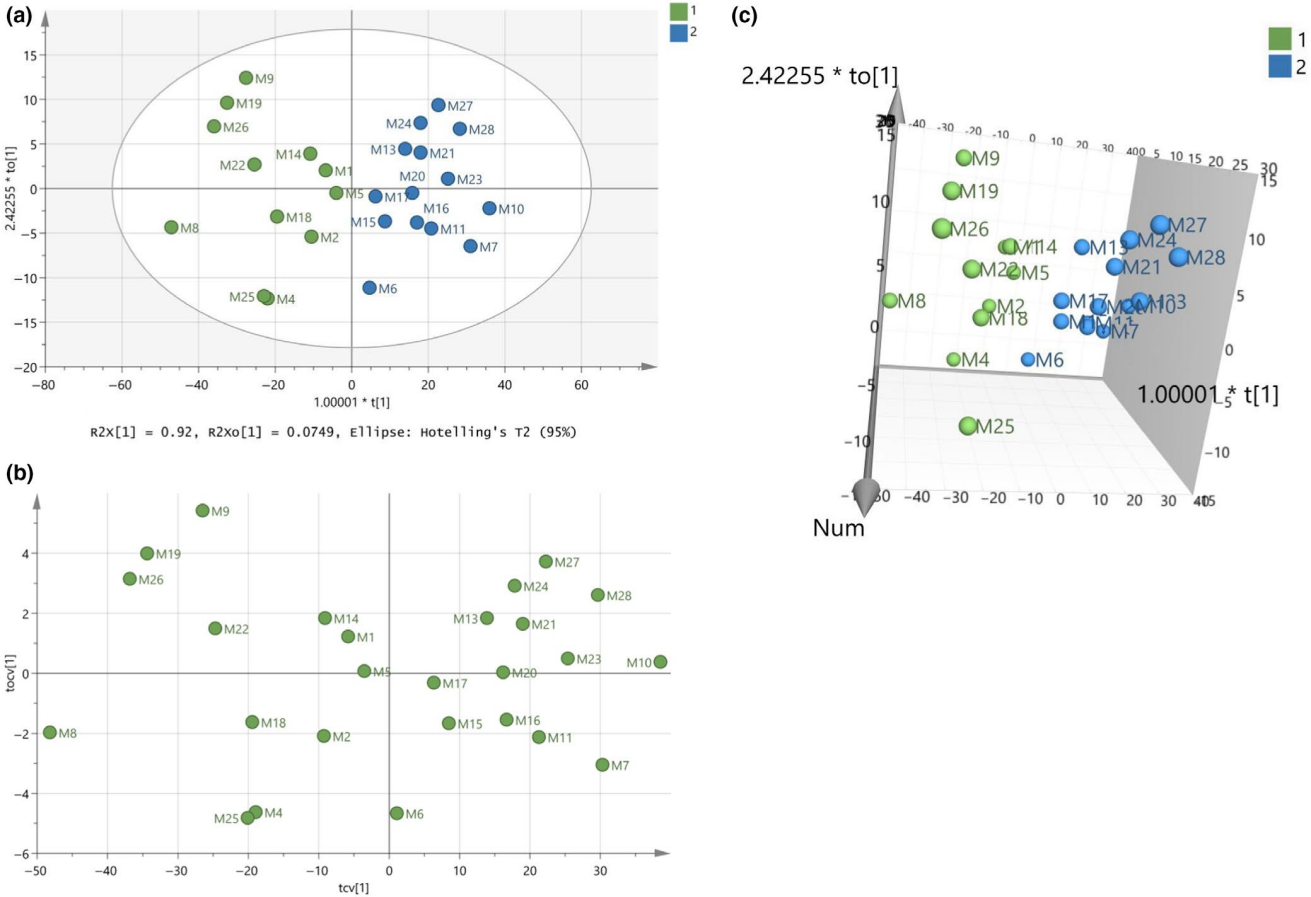
(a) Score scatter plot (to1/t1) from OPLS‐DA modeling of the final database regarding species’ origin of milk samples. A = 1+1 components, R^2^X(cum) = 0.995, R^2^Y(cum) = 0.791, Q^2^(cum) = 0.746, (b) CV score plot of the OPLS‐DA modeling, and (c) 3D presentation of the OPLS‐DA modeling

It is important to finalize a good database which can be considered as an authentic database of milk samples for future predictions. Subsequently, the model in Figure 6a should be optimized and validated. At this point, classes have been set for all the samples and Figures 7 and 8 indicate the final model constructed with PCA and OPLS‐DA, respectively. The samples have the same positions comparing the two graphs with small differences of the distances between them in some cases. The R^2^ and Q^2^ close to 1 indicated the model is an excellent fit. Table 3 shows that the OPLS‐DA model of the final database (Figure 8a,c) regarding species’ origin has 100% correct classification. None of the samples in Figure 8a showed to cross over to the other side in the CV score plot (Figure 8b), meaning that the constructed model is successful.

**TABLE 3:** Misclassification table from OPLS‐DA modeling of the final database (Figure 8) regarding species’ origin of milk samples, where 1: Cows samples and 2: Goats and sheep samples

	Members	Correct	1	2
1	12	100%	12	0
2	14	100%	0	14
Total	26	100%	12	14
Fisher's prob.	1e−07			

**FIGURE 9:**
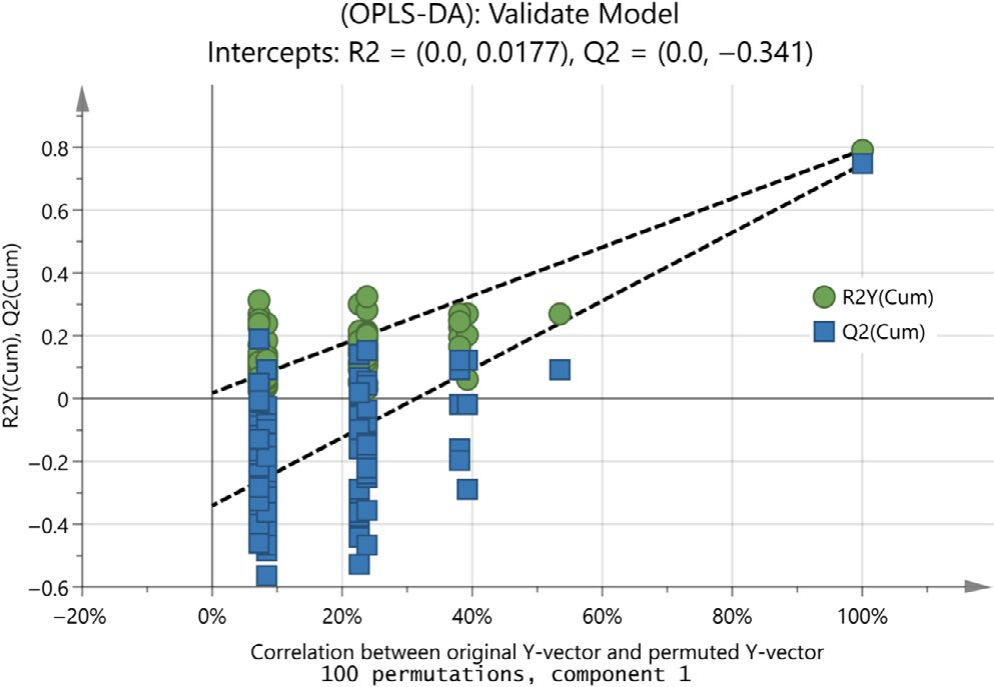
Random permutation test with 100 permutations for the final OPLS‐DA model in Figure 8 with R^2^X(cum) = 0.995, R^2^Y(cum) = 0.791, Q^2^(cum) = 0.746

Moreover, to validate the goodness of fit and the predictability of these results, a random permutation test with 100 permutations was employed, as seen in Figure 9. The criteria for validity that both R^2^ (original model) and Q^2^ (predictive model) located at right and permutated R^2^ (original model) and Q^2^ (predictive model) located left and all blue Q^2^ values to the left and right are lower than the green original R^2^ values. All the permuted models showed lower R^2^Y values if compared with the original model's R^2^Y value (0.791) and the majority of the Q^2^ regression lines showed negative intercepts (0.0, −0.323).

The ANOVA analysis showing the value of the *F*‐statistic and p‐value are depicted in Table 4. The model is highly significant, due to the *p*‐value of 5e‐06. The null hypothesis should be rejected, and the alternate hypothesis is considered important due to that *F*_statistic_ = 15.4 > *F*_critical_ =4.24.

**TABLE 4:** CV‐ANOVA results calculated for the OPLS‐DA model in Figure 8, where SS = sum of squares, DF = degree of freedom, MS = mean squares, p = *p*‐value of the test, *F* = *F* test calculated value or *F*_statistic_, and *SD* = standard deviation

	SS	DF	MS	F	p	*SD*
Total corr.	25	25	1			1
Regression	18.6	4	4.7	15.4	5e−06	2.2
Residual	6.4	21	0.3			0.6

### 3.4 Combination of the milk database with Halloumi cheese samples

**FIGURE 10:**
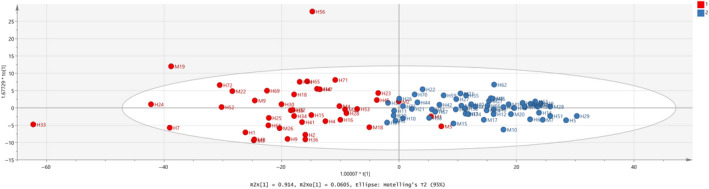
Score scatter plot (t2/t1) from OPLS‐DA modeling of Halloumi cheese and milk samples regarding species’ origin by using the subregion 1,150–400 cm^−1^ from FTIR spectra. A = 1 + 1 components, R^2^X(cum) = 0.974, R^2^Y(cum) = 0.686, Q^2^(cum) = 0.659

**FIGURE 11:**
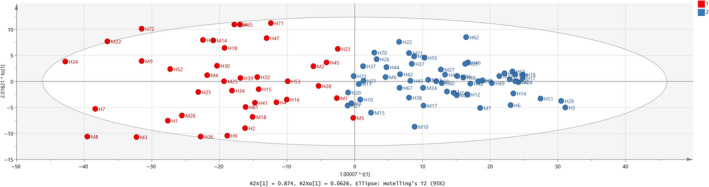
Score scatter plot (t2/t1) from OPLS‐DA modeling of Halloumi cheese and milk samples regarding species’ origin by using the subregion 1,150–400 cm^−1^ from FTIR spectra. A = 1 + 2 components, R^2^X(cum) = 0.977, R^2^Y(cum) = 0.740, Q^2^(cum) = 0.717. Red observations: Cows samples, Blue observations: Goats and sheep samples

**FIGURE 12:**
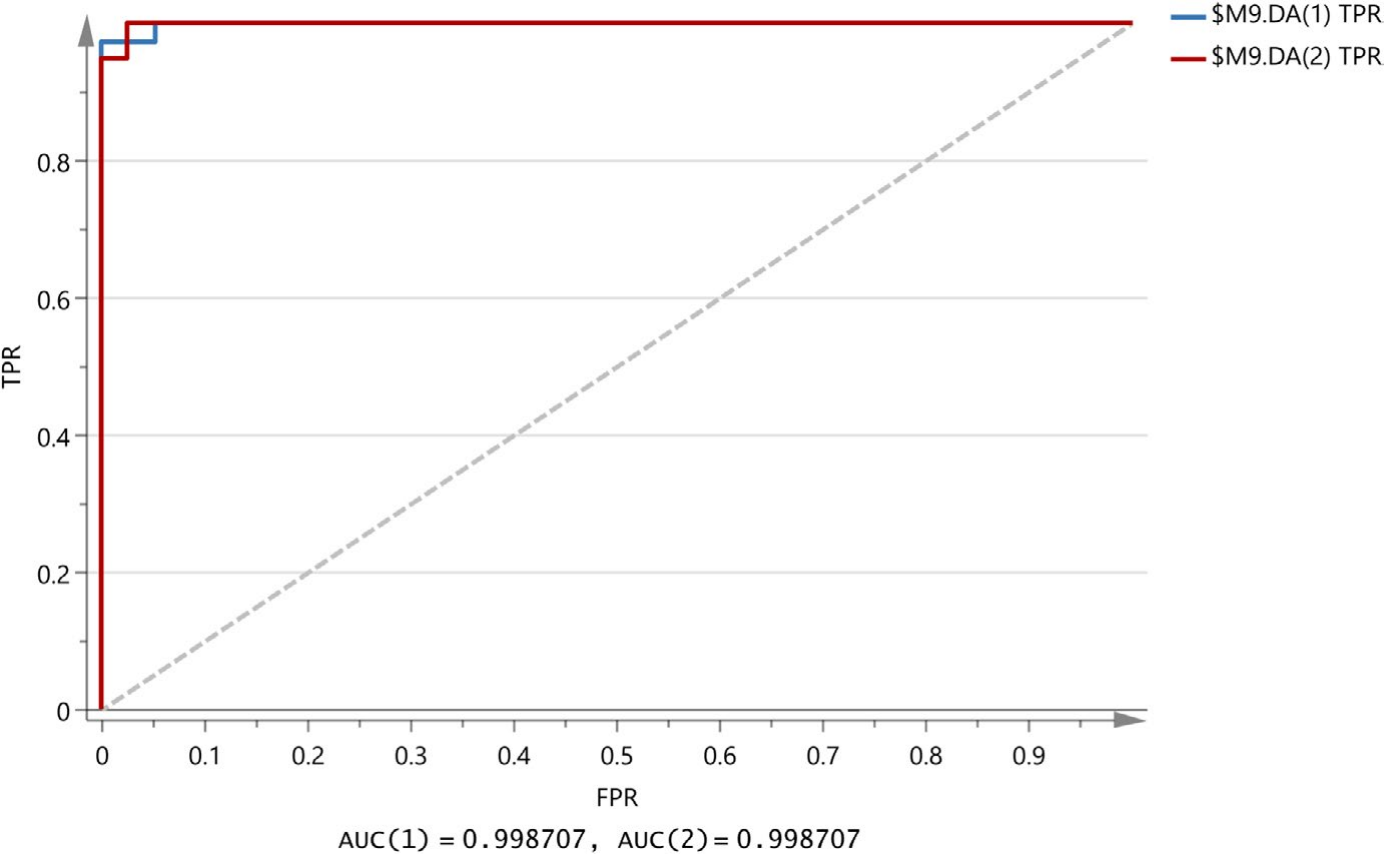
ROC curve for the OPLS‐DA model in Figure 11

Combination of the milk database with Halloumi cheese samples was initially tested with PCA method. A loading plot (not shown here) showed that only variables 1–390 should be included in the analysis which correspond to 1,150–400 cm^‐1^ from the FTIR values, which contains absorptions due to –NH_2_ deformation, COH bending, and C–C stretching with the contribution from OH bending and O = P–O (phosphate groups stretch) covalently bound to casein proteins and ‐C = O from polysaccharides and C = C stretching of acids. As a result, the model in Figure 10 was constructed with validation values R^2^X(cum) = 0.974, R^2^Y(cum) = 0.686, and Q^2^(cum) = 0.659. In addition, Table 5 shows that three samples are outliers and also 97.03% correct classification of the samples in the two classes. Finally, removal of the outlier samples, that is, M19, H33, and H56 showed a clear improvement of the OPLS‐DA model in Figure 11 with values R^2^X(cum) = 0.977, R^2^Y(cum) = 0.740, and Q^2^(cum) = 0.717. It can be said that the samples seem to be overlapped are milk samples, that is, M1 and M5, and that all Halloumi samples are well classified regarding the classification outlined in Table 6. The difference of the values R^2^X(cum) − Q^2^(cum) is 0.26 (less than 0.3), indicating that the model is good with high predictive ability. Table 7 shows a stable correct classification of samples to 97%. In Figure 12, ROC curve of the model shows that a good model was constructed with AUC equal to 0.999 for both classes.

**TABLE 5:** Misclassification table from OPLS‐DA modeling (Figure 10) of Halloumi cheese and milk samples regarding species’ origin by using the subregion 1,150 – 400 cm^‐1^ from FTIR spectra, where 1: Cows samples and 2: Goats and sheep samples

	Members	Correct	1	2
1	43	93.02%	40	3
2	58	100%	0	58
Total	101	97.03%	40	61
Fisher's prob.	5.4e−25			

**TABLE 6:** Misclassification table from OPLS‐DA modeling (Figure 11) of Halloumi cheese and milk samples regarding species’ origin for all the data obtained from FTIR spectra, where 1: Cows samples and 2: Goats and sheep samples

	Members	Correct	1	2
1	39	92.86%	36	3
2	58	100%	0	58
Total	97	97%	36	61
Fisher's prob.	1.3e−24			

**TABLE 7:** CV‐ANOVA results calculated for the OPLS‐DA model in Figure 11, where SS = sum of squares, DF = degree of freedom, MS = mean squares, p = *p*‐value of the test, *F* = *F* test calculated value or *F*_statistic_, and *SD* = standard deviation

	SS	DF	MS	F	p	*SD*
Total corr.	99	99	1			1
Regression	64.3	4	16.1	44	7.5e−21	4
Residual	34.7	95	0.4			0.6

**FIGURE 13:**
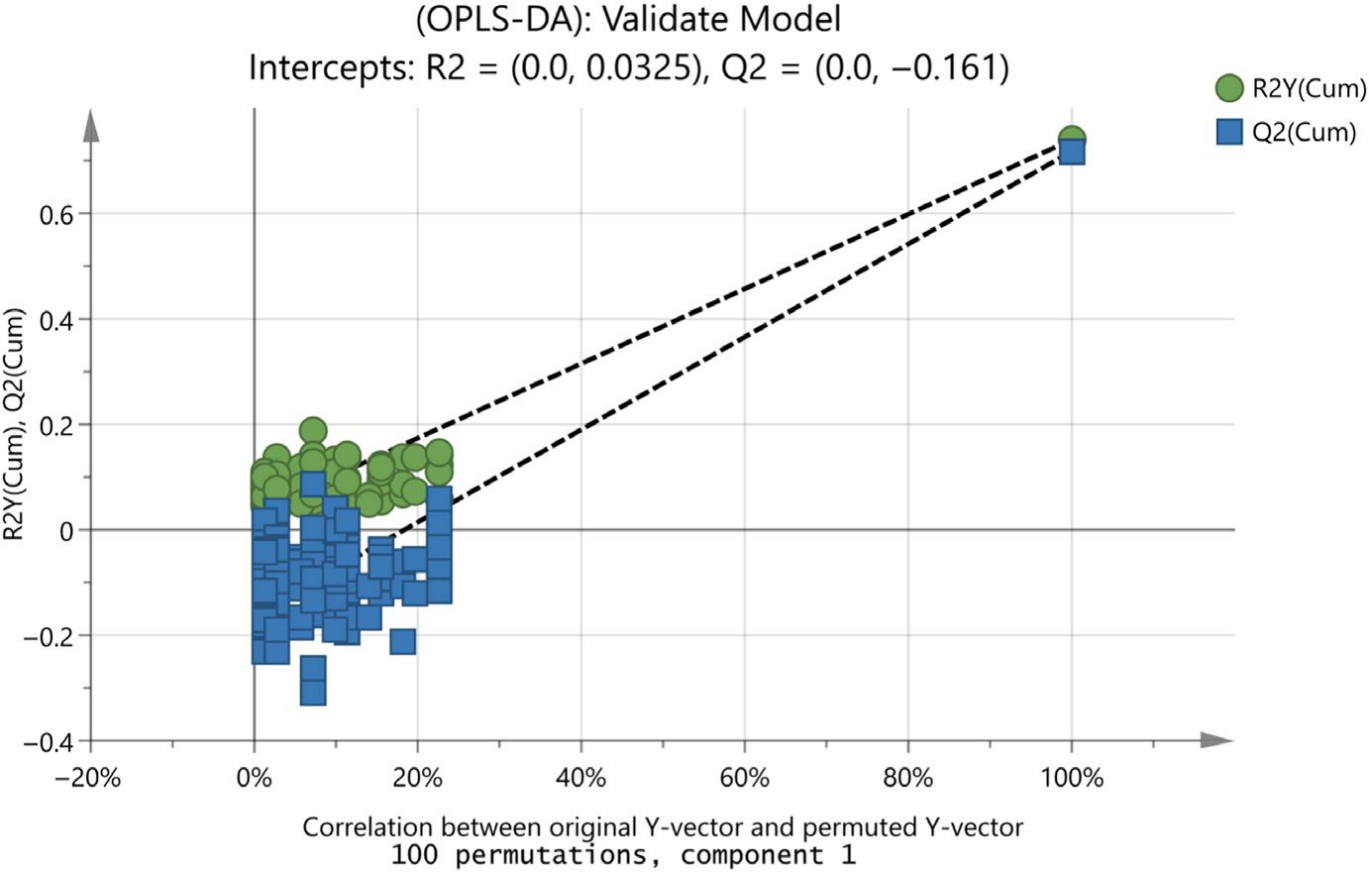
Random permutation test with 100 permutations for the OPLS‐DA model in Figure 11

A random permutation test with 100 permutations for the OPLS‐DA model in Figure 11 took place and it is presented in Figure 13. The criteria for validity include the following: all blue Q^2^ values to the left being lower than the original points to the right and the regression line of the Q^2^ points intersects the vertical axis at, or below zero. The R^2^ values always demonstrate some degree of confidence, although when all R^2^ points are lower than the original point to the right it also indicates a valid model (Eriksson et al., 2006). All the permuted models showed lower R^2^Y values if compared with the original model's R^2^Y value (0.74) and the majority of the Q^2^ regression lines showed negative intercepts (0.0, −0.167). The ANOVA analysis showing the value of the *F*‐statistic and p‐value are depicted in Table 7. The model is highly significant, due to the *p*‐value of 7.5e−21. The null hypothesis should be rejected, and the alternate hypothesis is considered important due to that *F*_statistic_ = 44> *F*_critical_ = 3.92–4.00. The model can serve as a validated database for future use.

The traditional recipe of Halloumi cheese demands the use of goat and/or sheep milk; however, fully cow milk is used by the large dairy industry. Low levels of production of goat and sheep milks reinforce this consequence, as well as cow milk is all year‐round available and has of course lower cost (Borkova & Snaselova, 2005; Pellegrino, Cattaneo, Masotti, & Psathas, 2010). Therefore, it is really important the fact that this research study managed to develop a method capable of determining and identifying the adulteration in Halloumi cheese due to mislabeling regarding species’ origin of milk.

## 4 CONCLUSIONS

The data enabled the differentiation of milk samples regarding species’ origin. The classification was satisfactory although similar variables existed among the samples. The peaks at wavenumbers 3,500–3,300 and 2,900–2,800 cm^−1^ were significant to predict the species’ origin of milk samples, and they correspond to ‐OH stretching in hydroxyl groups and C‐H bending in fatty acids, respectively. When the milk samples combined with Halloumi cheese samples, the subregion 1,150–400 cm^−1^ was only important for discrimination regarding species’ origin, which contains absorptions due to –NH_2_ deformation, COH bending, and C–C stretching with the contribution from OH bending and O = P–O (phosphate groups stretch) covalently bound to casein proteins and ‐C = O from polysaccharides and C = C stretching of acids.

The results augur well for the use of chemometric methods in the characterization of dairy products. Good results were obtained using both PCA and OPLS‐DA. OPLS‐DA appeared to have applicability for the chemometric analysis of the dairy products, since the total correct classification ability was very high as shown in misclassification tables and other validation tools. The FTIR spectroscopy technique coupled with chemometrics seems to be a powerful tool for controlling the authenticity of Halloumi.

The model constitutes an interesting approach and a preliminary work for future predictions of unknown Halloumi cheese samples regarding species’ origin. In the future, this model will also be studied regarding geographical origin of the samples. The proposed methodology contributes to adding extra value to Cypriot traditional Halloumi cheese.

## AUTHOR CONTRIBUTIONS

The experiments, including the Chemometric analysis, and the first draft of this paper were carried out by MT. RK performed the first Chemometric evaluation of the study. CRT edited and rewrote the first draft and produced the final paper. CRT also determined and refined the methodology, contributed to result interpretation, and provided overall supervision.

## ETHICAL STATEMENT

There are no conflicts of interest regarding the work reported herein. This study does not involve human or animal subjects.
